# Transcriptome and Metabolome Analyses Reveal Molecular Responses of Two Pepper (*Capsicum annuum* L.) Cultivars to Cold Stress

**DOI:** 10.3389/fpls.2022.819630

**Published:** 2022-03-22

**Authors:** Jianwei Zhang, Le Liang, Yongdong Xie, Zhao Zhao, Lihong Su, Yi Tang, Bo Sun, Yunsong Lai, Huanxiu Li

**Affiliations:** ^1^College of Horticulture, Sichuan Agricultural University, Chengdu, China; ^2^Institute for Processing and Storage of Agricultural Products, Chengdu Academy of Agricultural and Forest Sciences, Chengdu, China; ^3^Institute of Pomology and Olericulture, Sichuan Agricultural University, Chengdu, China

**Keywords:** pepper, transcriptomic, metabolome, cold stress, polyamines, ICE-CBF-COR

## Abstract

Low temperature is a significant factor affecting field-grown pepper. The molecular mechanisms behind peppers’ response to cold stress remain unknown. Transcriptomic and metabolomic analyses were used to investigate the responses of two pepper cultivars, XS (cold-sensitive) and GZ (cold-resistant), to cold stress; these were screened from 45 pepper materials. In this study, compared with the control group (0 h), we identified 10,931 differentially expressed genes (DEGs) in XS and GZ, 657 differentially expressed metabolites (DEMs) in the positive ion mode, and 390 DEMs in the negative ion mode. Most DEGs were involved in amino acid biosynthesis, plant hormone signal transduction, and the mitogen-activated protein kinase (MAPK) signaling pathway. Furthermore, metabolomic analysis revealed that the content of free polyamines (PAs), plant hormones, and osmolytes, mainly contained increased putrescine, spermine, spermidine, abscisic acid (ABA), jasmonic acid (JA), raffinose, and proline, in response to cold stress. Importantly, the regulation of the ICE (inducer of CBF expression)-CBF (C repeat binding factors)-COR (cold regulated) pathway by Ca^2+^ signaling, MAPK signaling, and reactive oxygen species (ROS) signaling plays a key role in regulating responses of peppers to cold stress. Above all, the results of the present study provide important insights into the response of peppers to cold stress, which will reveal the potential molecular mechanisms and contribute to pepper screening and breeding in the future.

## Introduction

Low temperature is a common abiotic stress in the natural environment, which has an important effect on growth and development, as well as the geographic distribution of crop plants. Cold stress can be divided into chilling (0–15°C) and freezing (<0°C) stress based on temperature. Generally, plants from temperature regions exposed to low, non-freezing temperatures for a period of time could increase their freezing tolerance in a process known as cold acclimation (CA; Chinnusamy et al., 2007). However, many valuable crops, such as rice, tobacco, tomato, and pepper, which originate in the tropics and subtropics, are sensitive to cold stress and incapable of CA (Yang et al., 2020). The first physical changes in plants under low temperatures are changes in the cell membrane (Yamada et al., 2002). When subjected to cold stress, the fluidity and structure of membrane proteins change, resulting in ionic imbalance, metabolic disorder, and even death (Yadav, 2010; Guo et al., 2018).

To minimize damage and ensure the healthy functioning of cells, plants have evolved a series of adaptive mechanisms. The cold regulation pathway ICE (inducer of CBF expression)-CBF (C repeat binding factors)-COR (cold regulated) is the best-understood defense mechanism in response to cold stress (Wang et al., 2017). Among them, CBFs are the most important transcription factors (TFs) that regulate cold stress. Although there are four CBF genes in *Arabidopsis*, only CBF1, CBF2, and CBF3, also known as *DREB1b* (dehydration response element binding factor 1b), *DREB1c*, and *DREB1a*, respectively, participate in cold regulation. CBF4 responded to drought and salt stress but not to low temperatures (Haake et al., 2002). All CBF genes belong to the apetala 2/ethylene response factor (AP2/ERF) family that participate in cold regulation. Furthermore, signaling molecules such as intracellular Ca^2+^, abscisic acid (ABA), and reactive oxygen species (ROS) play important roles in cold stress regulation (Chinnusamy et al., 2010). Calcium response elements recognize Ca^2+^ signatures and then transduce these signatures into downstream effects, including mediated phosphorylation events and altered gene expression patterns (McAinsh and Pittman, 2009; Heidarvand and Maali Amiri, 2010). There are two assumptions regarding the change in Ca^2+^ under stress: (1) damage to plant membranes results in ion leakage that induces changes in Ca^2+^ and (2) the accumulation of ROS triggered by stress leads to Ca^2+^ transients in plant cells (Yuan et al., 2018). Zhang et al. (2021) reported that respiratory burst oxidase homologs (Rbohs) are crucial for ROS production in plants. Of the seven members (*CaRbohA*–*CaRbohE*) identified in pepper, only *CaRbohA* and *CaRbohB* respond to cold stress.

Hundreds of metabolites have been identified that participate in the regulation of growth and development in plants under cold stress. For example, a variety of soluble sugars, such as glucose, fructose, raffinose, sucrose, and stachyose, are known to be associated with cold response, as they not only act as osmoprotectants but also function in plasma membrane integrity and osmotic balance in stressed plants (Ma et al., 2009; Sami et al., 2016). Amino acids play an essential role in synthesizing biologically active substances (Bryan et al., 2011) and act as precursors of a large number of metabolites with multiple functions (Krasensky and Jonak, 2012). Polyamines, which includes putrescine (Put), spermidine (Spd), and spermine (Spm), are involved in the regulation of plant responses under cold stress. In potatoes, arginine decarboxylase 1 (*ADC1*) expression increased in parallel with Put content and this increase could be essential for freezing tolerance (Kou et al., 2018). Conversely, *AtADC1/2* mutants with reduced Put content showed decreased freezing resistance in *Arabidopsis* (Cuevas et al., 2008).

High-throughput multi-omics technologies have made it possible to clarify the processes of plant responses to cold treatments. In *Nicotiana tabacum*, differences exist in energy metabolism and hormone metabolism between cold-resistant and cold-sensitive cultivars (Jin et al., 2017). A study on kiwifruit showed that the codeinone reductase and chalcone isomerase genes, nucleotide metabolism, and phenolic acid metabolism pathways participate in the regulation of freezing tolerance (Sun et al., 2021). Furthermore, Zhao Y. et al. (2019) demonstrated that differentially expressed genes (DEGs) and differentially expressed metabolites (DEMs) in wheat were significantly enriched in abscisic acid/jasmonate (ABA/JA) signaling and proline biosynthesis under cold stress. Raza et al. (2021) revealed that starch, sucrose, and amino acid metabolism regulated cold stress responses in rapeseed. Bai et al. (2021) found that the content of organic acids, sugars, and phenols, as well as cell wall metabolism, play an important role during cold storage of tomato fruits. Therefore, the application of multi-omics could be considered a fascinating method to preliminarily discover the key cold-responsive genes and mechanisms at the molecular levels.

Pepper (*Capsicum annuum* L. Solanaceae) originated in the tropical areas of Central and South America. It dominates today’s spice trade, and the global cultivation area has steadily increased, reaching approximately 3,800,000 ha in 2017 (Lin et al., 2013; Kang et al., 2020). As a thermophilic crop, cold stress is a major threat to pepper planting and production. Previous studies on cold stress and pepper have mainly focused on the physiology, TFs, and some functional genes (Guo et al., 2012; Hou et al., 2020). To the best of our knowledge, the molecular basis of cold stress in pepper has not yet been reported. In this study, transcriptome and metabolome analyses were carried out on the leaves of two pepper genotypes, XS (cold-sensitive) and GZ (cold-resistant), under control (0 h) and cold stress conditions. The aim was to reveal key genes and crucial metabolic pathways in response to cold stress between these contrasting cultivars. This study aims to provide important insights into the mechanisms underlying cold adaptation and tolerance in pepper plants.

## Materials and Methods

### Pepper Plants and Cold Treatments

The perennial GanZi plant (GZ, a cold-resistant cultivar) and the annual XianSheng plant (XS, a cold-sensitive cultivar) were used for the experiments. The pepper seeds were sown in a 50-hole plug tray containing mixed substrates of peat: vermiculite: perlite (2:1:1, v/v/v) in a greenhouse at 25°C (16 h/day) and 20°C (8 h/night) until four true leaves developed. Seedlings with uniform growth were selected and transferred to pots (9 cm × 9 cm). The seedlings were grown to the six-leaf stage and then subjected to cold stress at 4°C. Leaf samples were harvested at 0, 6, and 24 h. Physiological indexes and transcriptomic experiments were performed with three biological replicates per treatment. Metabolomics experiments were carried out with six biological replicates. All the samples were frozen in liquid nitrogen and stored at −80°C for follow-up tests.

### Physiological Measurements of Pepper Seedlings

The relative electrolyte conductivity (REC) and proline content were determined according to the method of Geng et al. (2019). Malondialdehyde (MDA) and superoxide dismutase (SOD) were determined in the samples using plant MDA assay kit and total SOD assay kit (Nanjing Jiancheng Bioengineering Institute, Nanjing, China), respectively, following the manufacturer’s instructions. Chlorophyll content was determined following the method described by Brini et al. (2009). Statistical calculations were performed using SPSS 17.0, and Duncan’s test was used for significance analysis (*P* < 0.05, significant; *P* < 0.01, highly significant).

### cDNA Library Construction, Sequencing, and Data Analysis

Exactly 0.6 g of each of the 18 leaf samples was preserved in dry ice and sent to Biomarker Technologies (Beijing, China) for library construction, quality control, and paired-end sequencing with Illumina HiSeq. After cDNA library sequencing, many high-quality raw reads were selected, and clean reads were obtained by removing low-quality ones. All the clean reads were mapped separately to the “Zunla1” pepper genome^1^ assembly using HISAT2 software. The unigenes were compared to public databases [Non-redundant Protein Database (NR), Gene ontology (GO), Kyoto Encyclopedia of Genes and Genomes (KEGG), Kyoto Encyclopedia of Genes and Genomes (KOG), Clusters of Orthologous Groups (COG), Protein Family Database (Pfam), and Non-redundant Protein Sequence Database (Swiss-Prot)] using BLAST with an *e*-value threshold of 10^–5^.

### Identification and Analysis of Differentially Expressed Genes

To verify the transcription expression levels of all samples, fragments per kilobase of transcript per million mapped reads (FPKM) were used to quantify the expression level of genes. Subsequently, DEGs between control samples and cold-treated samples were identified using DESeq2 software, with |log2 fold change (FC)| ≥ 1 and false discovery rate (FDR) <0.01. DEGs of XS and GZ at different time stages were clustered based on the short time-series expression miner (STEM) cluster method (Ernst and Bar-Joseph, 2006). GO and KEGG pathway enrichment analyses were performed using the BMK Cloud platform^2^. The heat map of DEGs was constructed using the TBtools software (Chen et al., 2020).

### Quantitative Real-Time Polymerase Chain Reaction Verification

The accuracy of the transcriptome data was verified using qRT-PCR. Total RNA from the leaves, for each treatment, was extracted using the Plant RNA Extraction Kit (TaKaRa, Japan) according to the manufacturer’s instructions. The RNA concentration was determined using a spectrophotometer (Thermo Fisher Scientific Oy, Finland), and cDNA synthesis was performed using the Bio-Rad iScript cDNA synthesis kit (TaKaRa, Japan). Eleven DEGs were randomly selected to determine their expression levels. The primers for these genes were designed by Primer3web^3^, and NCBI database was used to detect their specificity. qRT-PCR was conducted using 2X SYBR Green Fast qPCR Mix (Biomarker, China) on a CFX96 real-time PCR system (Bio-Rad, United States). The relative expression was calculated by the 2^–ΔΔct^ method (Livak and Schmittgen, 2001) and the *CaUbi3* (Accession No. AY486137) was used as the reference gene (Yin et al., 2014). All primer sequences are listed in Supplementary Table 1.

### Metabolite Profiling

A total of 36 leaf samples were used for determining the untargeted metabolites based on the liquid chromatography with tandem mass spectrometry (LC-MS/MS) platform (Biomarker Technologies Co., Ltd.). Exactly 0.2 g of the sample was added to 1 mL extraction liquid (methanol: acetonitrile: water = 2:2:1) and 2 μL internal standard (2-Chloro-L-phenylalanine). The samples were homogenized in a ball mill and ultrasonicated in ice water for 5 min. After three further homogenization steps, the samples were incubated for 1 h at −20°C to precipitate the proteins. Thereafter, the samples were centrifuged at 12,000 × *g* for 15 min at 4°C, and 0.2 mL of the supernatant was transferred to the LC-MS vials. Finally, 120 μl sample was taken for UHPLC-QE orbitrap/MS analysis. LC-MS/MS analyses were performed on a Waters ACQUITY UPLC I-Class PLUS system (Waters Corp., Milford, CT, United States). An Acquity UPLC HSS T3 column (Waters Corp., Milford, CT, United States) was used in this study. The samples were analyzed in both the positive and negative ion modes. MS raw data were collected using MassLynx software (version 4.2, Waters Corp., Milford, CT, United States) and processed by the Progenesis QI software (Waters Corp., Milford, CT, United States). The Metlin database (Waters Corp., Milford, CT, United States) and an in-house database (Biomarker Technologies Co., LTD.) were used for peak annotation and identification. Additionally, DEMs were extracted based on the values retrieved by variable importance in the projection (VIP > 1), Student’s *t*-test (*p* < 0.05), and |log2FC| ≥ 0.58. The DEM analysis method is similar to that of DEGs.

### Integrative Analysis of Transcriptome and Metabolome

Genes and metabolites with a Pearson correlation coefficient (PCC) >|0.8| and *P*-value < 0.05, were used to establish the related network, which was visualized using the Cytoscape software (Shannon et al., 2003).

### Determination of Polyamine Content and Enzyme Activity

Free polyamines (Put, Spd, and Spm) in leaf tissues were determined as described by Zhou et al. (2019). Briefly, 0.5 g leaf samples were homogenized in 3 mL of 5% perchloric acid and incubated at 4°C for 1 h, followed by centrifugation at 13,000 × *g* for 25 min at 4°C. Exactly 0.5 mL of the supernatant was mixed with 10 μL of benzoyl chloride and 1 mL of 2 M NaOH in a plastic tube, and incubated for 20 min at 37°C. After incubation, 2 mL of saturated NaCl and 2 mL of dimethyl ether were added to the mixture and centrifuged at 5,000 × *g* for 8 min. One milliliter of the ether phase was evaporated to dryness. Finally, 1 mL of methanol was added to the centrifuge tube and filtered through a 0.45-μm membrane. The injection volume was 10 μL. The mobile phase was 70% methanol, and the flow rate was 0.7 mL/min. The PA peaks were detected at 230 nm. The standard sample (Put, Spd, and Spm) was purchased from Sigma-Aldrich (Dallas, TX, United States) and the standard calibration curves were constructed according to Lin et al. (2016).

The activities of ADC, ornithine decarboxylase (ODC), spermidine synthase (SPDS), and polyamine oxidase (PAO) were determined using a plant ELISA (enzyme linked immunosorbent assay) kit (Chundu Biotechnology, Wuhan, China) following the manufacturer’s recommendations.

## Results

### Phenotypic Differences Between GanZi and XianSheng in Response to Cold Stress

**FIGURE 1:**
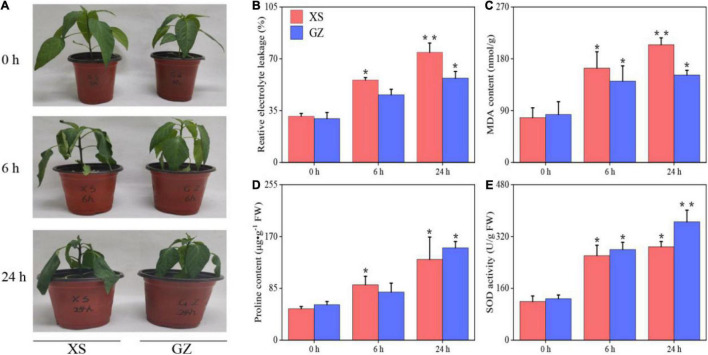
**(A)** Morphological changes in the cold-sensitive (XS) and cold-tolerant (GZ) cultivars in response to cold stress. **(B)** Relative electrolyte conductivity (REC), **(C)** MDA content, **(D)** proline content, **(E)** SOD activity. “*” means *P* < 0.05 between treatment and control; “**” means *P* < 0.01 between treatment and control.

Previously, we identified the cold-sensitive variety XS and cold-tolerant variety GZ from 45 pepper materials through physiological indexes (Supplementary Table 2). In this study, no differences were observed between the two cultivars exposed to control conditions. However, the XS plants showed leaf curling after 6 h of cold treatment, and the leaves wilted after 24 h. The GZ plants grew normally with no leaf symptoms after 24 h of cold stress (Figure 1A). In addition, we measured physiological indices of these two varieties after cold stress. As shown in Figures 1B,C, the REC and MDA of XS increased significantly (*P* < 0.05) after 6 h of cold stress, and the increases were highly significant (*P* < 0.01) after 24 h compared to the control. The REC and MDA of GZ increased significantly after 24 h compared to the control. The increment of REC of XS and GZ cultivars were 2.37-fold and 1.92- at 24 h, respectively, and that of MDA was 2.63- and 1.83- at 24 h compared to the control. Both the proline content and SOD activity showed an increasing trend than those in the control (Figures 1D,E). The increment of proline content and SOD activity in GZ (2.59- and 2.85-) was higher than that in XS (2.57- and 2.41-) after 24 h of stress. These results indicate that GZ is more tolerant to cold than XS.

### RNA Sequencing, Assembly, and Quantitative Real-Time Polymerase Chain Reaction Validation

A total of 18 samples (three biological replicates of six treatments) were processed, resulting in approximately 38.76–50.75 Mb raw reads (Table 1). After filtering, the clean reads were 19.38–25.37 Mb, and the length ranged from 5.79 to 7.58 Gb. The average GC content was 44.35%, and the Q30 of the clean reads exceeded 92.75%. The unique mapped ratio ranged from 78.70 to 85.34%, and the total mapped ratio was over 85.24%. The correlation between the three biological replicates was *R*^2^ > 0.84 for all treatments, except for sample XS62 (*R*^2^ < 0.8), which was discarded (Supplementary Table 3). In addition, 11 genes with different expression patterns were randomly selected for qRT-PCR. Most of the genes showed a consistent expression profile between qRT-PCR and RNA-seq (Supplementary Figure 1), and the correlation was measured by scatter plotting log2-fold changes (Supplementary Figure 2), which showed a positive correlation coefficient (*R*^2^ = 0.71). This suggested the RNA-seq data were of high quality and could be used for subsequent analyses.

**TABLE 1:** Summary of the RNA-seq data collected from cold-sensitive cultivar XS and cold-tolerant cultivar GZ.

Samples	Raw reads	Clean reads	Clean bases	GC content	% ≥Q30	Mapped ratio	Unique mapped ratio
XS01	40,281,138	20,140,569	6,015,747,870	44.79%	94.04%	91.94%	84.60%
XS02	50,747,678	25,373,839	7,583,956,216	44.57%	93.82%	92.30%	84.89%
XS03	44,202,314	22,101,157	6,598,588,766	44.88%	93.71%	90.57%	83.19%
XS61	42,560,338	21,280,169	6,351,321,880	44.71%	93.90%	92.33%	84.10%
XS62	41,722,806	20,861,403	6,230,353,866	44.99%	92.96%	89.35%	81.36%
XS63	39,923,908	19,961,954	5,959,784,526	44.52%	93.64%	91.42%	83.73%
XS241	44,613,652	22,306,826	6,674,149,186	43.71%	92.77%	90.24%	84.44%
XS242	43,713,956	21,856,978	6,500,773,330	44.20%	92.85%	89.78%	82.59%
XS243	45,150,502	22,575,251	6,748,540,814	43.45%	92.82%	91.27%	85.34%
GZ01	47,338,208	23,669,104	7,078,433,996	44.59%	93.21%	89.93%	82.74%
GZ02	48,399,988	24,199,994	7,228,212,928	45.28%	93.03%	87.33%	78.70%
GZ03	41,368,090	20,684,045	6,186,718,374	44.25%	93.63%	85.24%	79.89%
GZ61	45,930,906	22,965,453	6,857,008,448	44.06%	94.05%	92.18%	84.44%
GZ62	38,761,812	19,380,906	5,792,248,358	44.42%	93.85%	92.15%	84.43%
GZ63	45,259,532	22,629,766	6,759,081,298	44.21%	93.74%	90.37%	83.15%
GZ241	45,165,406	22,582,703	6,730,878,196	43.99%	92.83%	90.03%	83.47%
GZ242	44,138,600	22,069,300	6,597,811,096	43.70%	92.75%	90.16%	83.93%
GZ243	39,071,708	19,535,854	5,841,942,978	44.03%	93.46%	88.33%	81.69%

### Identification of Differentially Expressed Genes and Analysis of Expression Pattern

**FIGURE 2:**
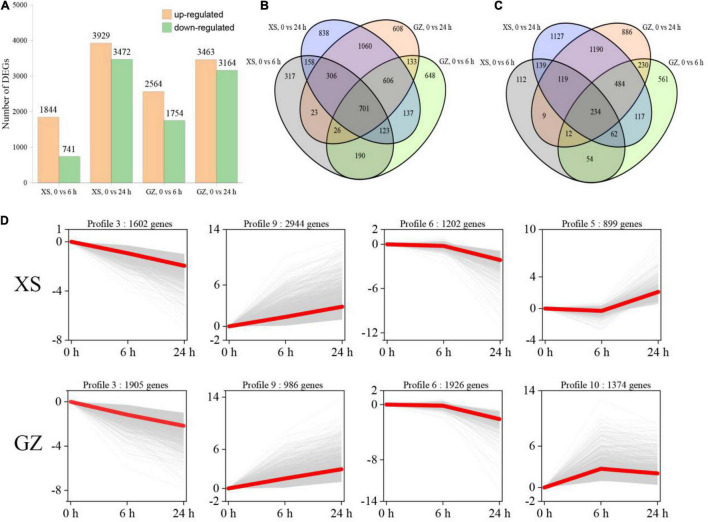
Gene expression profile of different pepper genotypes in response to cold stress. **(A)** Total number of upregulated and downregulated differentially expressed genes (DEGs). **(B)** Venn diagram of upregulated DEGs. **(C)** Venn diagram of downregulated DEGs. **(D)** Patterns of DEGs expressions across three time points in cold-sensitive cultivar XS and cold-tolerant cultivar GZ. In each frame, the gray lines represent the gene expression pattern, and the red lines represent the expression tendency of all the genes. The number of genes belonging to each pattern is given above the frame.

Based on the criteria of FDR < 0.01 and |log2FC| ≥ 1, we identified a total of 10,931 DEGs in the two cultivars after cold stress (Supplementary Table 4). There were 1,844 (upregulated)/741 (downregulated) in XS after cold stress at 6 h, and 3,929/3,472 DEGs at 24 h. A total of 2,564/1,754 and 3,463/3,164 DEGs were identified in the GZ at 6 and 24 h, respectively (Figure 2A). Venn diagrams showed common and unique DEGs between cold-treated and control transcriptomes (Figures 2B,C). In our study, the results showed that 701 upregulated DEGs and 234 commonly downregulated DEGs were obtained in the overlapping regions of the Venn diagram, respectively. The number of unique DEGs in GZ was greater (lower) than that of XS at 6 h (24 h), the results showed that GZ had more genes involved in cold regulation during the early stages of cold stress. DEGs of XS and GZ at different time stages clustered in 11 profiles based on the STEM cluster method, and the profiles with significant differences (*P* < 0.05) in XS and GZ were listed (Figure 2D). It was obvious that there were three identical expression profiles, including profiles 3, 9, and 6, in the two varieties, while profile 5 appeared only in XS and profile 10 only in GZ. The number of upregulated genes in XS (profiles 9 and 5) was greater than that in GZ (profiles 9 and 10), while the number of downregulated genes (profile 3 and profile 6) in GZ was greater than in XS. These results indicated that differential gene expression patterns in the two genotypes could be the basis for their different tolerances to cold stress.

### Functional Annotation of Differentially Expressed Genes

**FIGURE 3:**
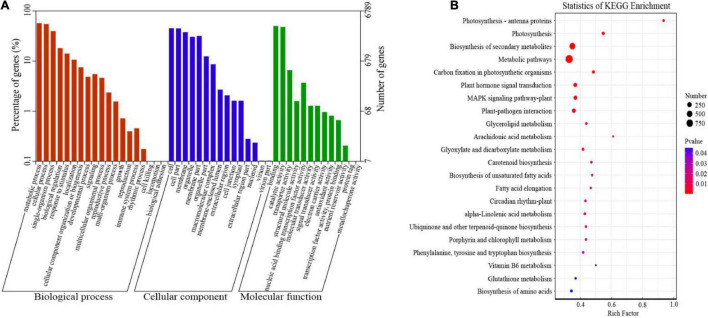
Functional annotations of differentially expressed genes (DEGs) in cold-stressed pepper leaves. **(A)** Gene ontology (GO) classification of DEGs. **(B)** Kyoto Encyclopedia of Genes and Genomes (KEGG) pathway enrichment and bubble chart.

Gene ontology and KEGG databases were used to explore the potential functions of all DEGs under cold stress. The 6,789 DEGs were categorized into 47 GO terms concerning biological process, cellular component, and molecular function categories (Figure 3A and Supplementary Table 5). The most abundant genes belonged to “metabolic processes” (3,879 genes), followed by “cellular processes” (3,723 genes), and “single-organism process” (2,703 genes). In the cellular component category, the number of genes in “cells” (3,080 genes) and “cell parts” (3,054 genes) was the highest. The primary terms of the molecular function category were “binding” (3,407 genes) and “catalytic activity” (3,270 genes). In terms of the KEGG analysis, a total of 4,656 DEGs were assigned to 132 KEGG pathways, and we listed 22 pathways based on the *P*-value (*P* < 0.05) (Figure 3B and Supplementary Table 6). Most of the pathways were grouped into “metabolic pathways” (160 genes) and “biosynthesis of secondary metabolites” (74 genes), followed by “plant hormone signal transduction” (130 genes), “MAPK signaling pathway” (124 genes), and “glutathione metabolism” (48 genes). In addition, some DEGs such as phenylalanine, tyrosine, and tryptophan biosynthesis clustered into the “biosynthesis of amino acids” (99 genes) category. The above results showed that all these pathways play a key role in pepper response to cold stress.

### Analysis of Genes Encoding Transcription Factors

**FIGURE 4:**
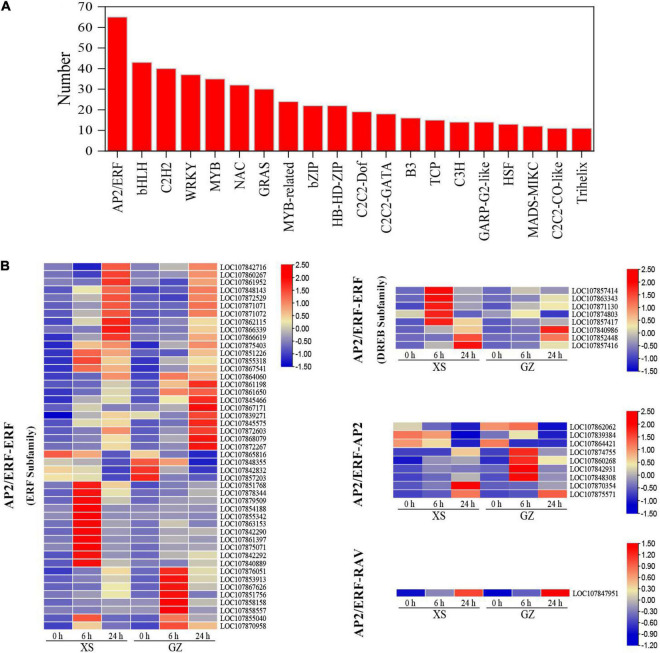
Analysis of transcription factors (TFs) in differentially expressed genes (DEGs). **(A)** Top 20 transcriptome factors. **(B)** Classification of AP2/ERF family. Color bins represent Log10 FPKM of a gene.

Transcription factors play important roles in regulating the expression of stress-responsive genes under cold stress. In this study, we detected 625 differentially expressed TFs, which mainly belonged to AP2/ERF (65), bHLH (43), C2H2 (40), WRKY (37), MYB (35), NAC (32), and GRAS (30), comprising over 30 genes families (Figure 4A and Supplementary Table 7). The AP2/ERF family was divided into three groups, the ERF, AP2, and RAV, based on their repetitive structure. According to the different conserved amino acid sequences, the ERF family was further divided into ERF and DREB subfamilies. In all the tAP2/ERF members, we identified 47 ERF (ERF subfamily) members, 8 ERF (DREB subfamily) members, 9 AP2 members, and 1 RAV member (Figure 4B). In the ERF subfamily, some TFs were highly expressed after 6 h, while others were highly expressed after 24 h. For example, LOC107861397, LOC107855040, and LOC107863153 had 8.56/5. 70-, 5.61/5. 22-, and 3.77/2.25-fold change under cold stress in XS/GZ at 6 h, respectively. LOC107866339, LOC107868079, LOC107871071, and LOC107872603 had 8.75/8. 04-, 6.48/7. 94-, 6.81/5. 82-, and 5.96/6.25-fold change under cold stress in XS/GZ at 24 h, respectively. In the DREB subfamily, the expression of LOC107840986, LOC107852448, and LOC107857416 increased with treatment time. With regard to AP2/ERF-AP2 and AP2/ERF-RAV, LOC107860268 and LOC107848308 were highly expressed at 24 h in GZ. However, the expression of these two genes was highest at 6 h in XS. The expression of LOC107847951 in the two varieties increased significantly during cold stress and reached a maximum at 24 h.

### Metabolic Analysis of XianSheng and GanZi in Response to Cold Stress

Six repetitions were conducted for each treatment in the metabolic analysis to ensure the reliability of the experiment. In total, 1,101 metabolites were identified in the positive ion mode (Supplementary Table 8) and 574 metabolites in the negative ion mode (Supplementary Table 9) from both species. Among them, it contains 247 same metabolites in positive and negative ion mode.

In positive ion mode, according to the principal component analysis (PCA), the first PC (PC1) explained 40.6% of the variation, and there was an obvious separation between the XS and GZ species (Supplementary Figure 3A). DEMs were defined with a |log2FC| ≥ 0.58, *P* < 0.05, and VIP > 1. A total of 120 (upregulated)/99 (downregulated) and 179/214 metabolites were identified in XS at 6 and 24 h, respectively, and a total of 37/118 and 63/228 metabolites in GZ, respectively (Supplementary Figure 3B and Supplementary Table 10). The number of upregulated (downregulated) metabolites in XS was higher (lower) than that in GZ. In addition, the number of XS and GZ DEMs at 24 h was greater than that at 6 h. These results indicated that the 24 h cold treatment had a greater impact on the pepper transcriptomes than the 6 h treatment. The Venn diagram results show that 50, 118, 23, and 12 upregulated DEMs were unique to XS and GZ under cold stress, and 7 DEMs shared the same patterns (Supplementary Figure 3C). For downregulated DEMs, 36, 102, 102, and 32 DEMs were specifically expressed, and 4 DEMs shared the same patterns (Supplementary Figure 3D).

PC1 and PC2 accounted for 34.6 and 14.8% of the variation in the negative ion mode, respectively (Supplementary Figure 4A). In the statistical diagram of DEMs, a conclusion similar to that of the positive ion mode was obtained (Supplementary Figure 4B and Supplementary Table 11). The number of unique DEMs decreased in the negative ion mode than in the positive ion mode; only two were commonly upregulated and one was commonly downregulated (Supplementary Figures 4C,D).

### Kyoto Encyclopedia of Genes and Genomes Enrichment Analysis of Differentially Expressed Metabolites in Positive and Negative Ion Mode

**FIGURE 5:**
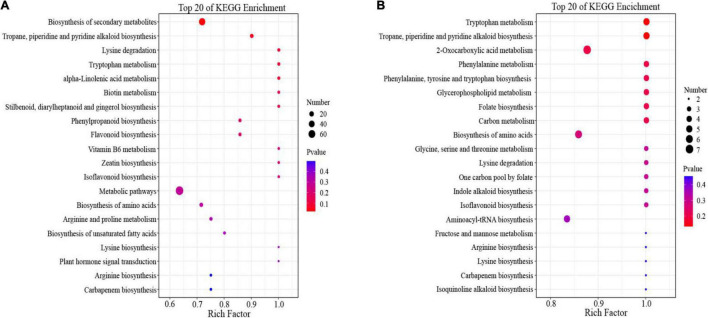
Functional Kyoto Encyclopedia of Genes and Genomes (KEGG) pathway classification of differentially expressed metabolites (DEMs) in **(A)** positive ion mode and **(B)** negative ion mode.

To better understand the functions of the DEMs, we mapped all DEMs into the KEGG pathway database. In the positive ion mode (Figure 5A and Supplementary Table 12), 654 DEMs were mainly enriched in “metabolic pathways,” “biosynthesis of secondary metabolites,” and the amino acid metabolic pathways, mainly included “lysine degradation,” “tryptophan metabolism,” “lysine biosynthesis,” “arginine and proline metabolism,” and “arginine biosynthesis.”

In the negative ion mode (Figure 5B and Supplementary Table 13), we found that most of DEMs were grouped into “2-Oxocarboxylic acid metabolism,” “biosynthesis of amino acid,” and “aminoacyl-tRNA biosynthesis.” interestingly, several metabolic pathways of amino acids were also enriched in negative ion mode; these included: “tryptophan metabolism,” “phenylalanine metabolism,” “lysine degradation,” “arginine biosynthesis,” “phenylalanine, tyrosine and tryptophan biosynthesis,” and “lysine biosynthesis.”

Combining the KEGG pathways of DEGs and DEMs, some common pathways for DEGs and DEMs, such as “plant hormone signal transduction,” “biosynthesis of amino acid,” “vitamin B6 metabolism,” “alpha-Linolenic acid metabolism,” and “biosynthesis of unsaturated fatty acids” were established. However, some pathways that played an important role in the regulation of cold stress were established only for DEGs or DEMs; these included “glutathione metabolism,” “carbon metabolism,” and “fructose and mannose metabolism.” We further analyzed its relationship with cold stress in pepper plants.

### Plant Hormone Pathway Response to Cold Stress

**FIGURE 6:**
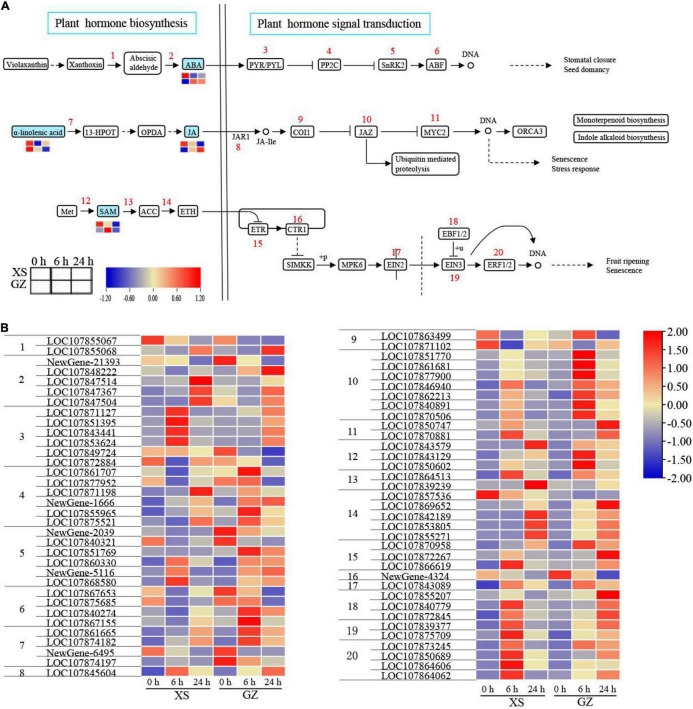
Adaptive changes involved in plant hormone biosynthesis and plant hormone signal transduction in cold-sensitive cultivar XS and cold-tolerant cultivar GZ under cold stress. **(A)** Metabolic changes in XS and GZ under cold stress. **(B)** Transcriptional changes of the genes in XS and GZ under cold stress. Each graph represents the normalized intensity of corresponding metabolite or genes in different sampling points.

As mentioned above, we found that many DEGs and DEMs were enriched in plant hormone biosynthesis and plant hormone signal transduction pathways; the hormones were mainly ABA, JA, and ethylene (ETH; Figures 6A,B). In plant hormone biosynthesis, four DEMs and 21 DEGs were identified to be involved in ABA, JA, and ETH biosynthesis pathways, while 48 DEGs were involved in hormone signal transduction.

In the ABA pathway, xanthoxin dehydrogenase converted xanthoxin to abscisic aldehyde, and interestingly, there were two genes (LOC107855067 and LOC107855068) were predicted to encode xanthoxin dehydrogenase that showed the opposite trend. Abscisic-aldehyde oxidase acts as a key enzyme in ABA synthesis and was upregulated in two genotypes, except for the Newgene-21393 gene being downregulated. Compared with control, the content of ABA (−, represents in negative ion mode) decreased in XS but increased in GZ. Pyrabaction resistant/PYR-like (PYR/PRL) is an ABA receptor of the signaling complex, with six genes encoding this protein. They were mainly (66%) upregulated in both genotypes. The expression of these genes increased only after 6 h in XS but after 24 h in GZ. The genes encoding 2C-type protein phosphatase (PP2C), SNF1-related protein kinase 2 (SnRK2), and ABA-responsive element binding factor (ABF), were upregulated in GZ at rates of 66, 66, and 50%, respectively.

In the JA pathway, α-Linolenic acid (+, represents in positive ion mode) was lower in GZ under cold stress than in plants under the control conditions. The content of JA (−) was observed to be strongly cold-induced in GZ under cold stress, whereas it decreased remarkably in XS plants, especially after 24 h. The genes encoding coronatine insensitive protein (COI1) were downregulated under cold stress, whereas the genes encoding another three proteins, jasmonic acid-amido synthetase (JAR1), jasmonate ZIM-domain (JAZ), and myelocytomatosis proteins 2 (MYC2), were upregulated in XS and GZ under cold stress.

Ethylene hormone biosynthesis predominantly involves three conversions: methionine (Met) to S-adenosyl methionine (SAM), SAM to 1-aminocyclopropane-1-carboxylic acid (ACC), and ACC to ETH. All the genes involved in the above steps were upregulated under cold stress in both genotypes, except for LOC107857536. For ETH signal transduction, genes encoding ethylene receptor (ETR), ethylene insensitive 2 (EIN2), ethylene-binding factor 1/2, ethylene insensitive 3 (EIN3), and ethylene-responsive factor 1/2 (ERF1/2) were upregulated at 6 or 24 h in the two genotypes. The expression of the CRT1 was downregulated in XS and GZ under cold stress. These results indicate that all DEGs and DEMs play an important role in regulating plant responses to cold stress.

### Sugar and Amino Acid Metabolism in Pepper Under Cold Stress

**FIGURE 7:**
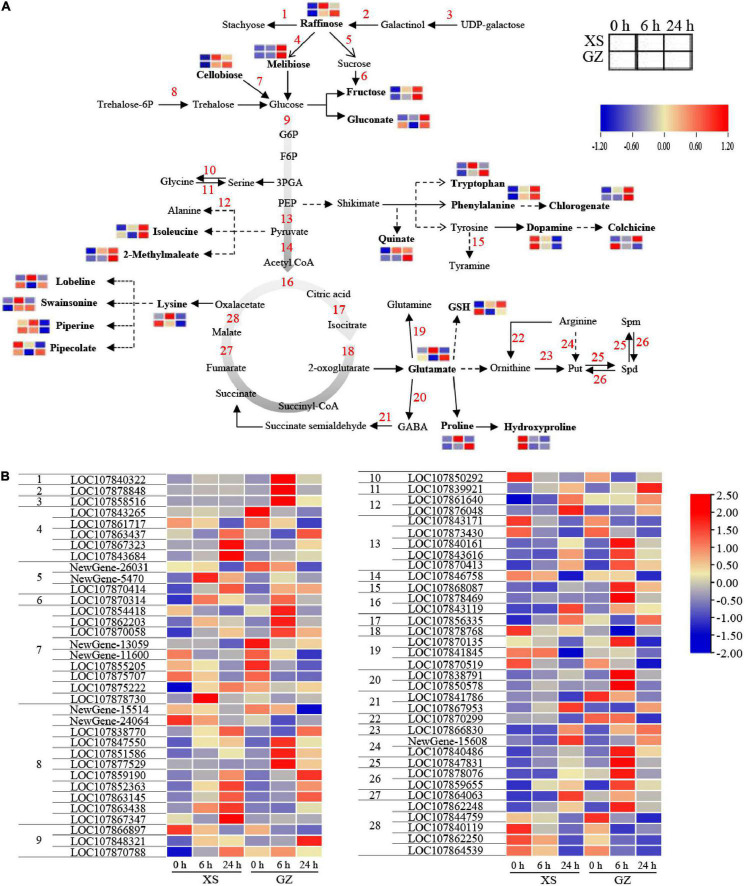
Adaptive changes involved in sugar and amino acid metabolic pathways in cold-sensitive cultivar XS and cold-tolerant cultivar GZ under cold stress. **(A)** Metabolic changes in XS and GZ under cold stress. **(B)** Transcriptional changes of the genes in XS and GZ under cold stress. Each graph represents the normalized intensity of the corresponding metabolite or genes at different sampling points.

According to our results, cold stress had a significant influence on amino acid metabolism, carbon metabolism, glutamate biosynthesis, and polyamine metabolism. Figure 7A shows that most of the metabolites involved in sugar metabolism, including raffinose (−), melibiose (−), cellobiose (−), and fructose (−), were upregulated under cold stress in XS and GZ, except for gluconate in GZ after 6 h. Several genes were predicted to encode stachyose synthetase, raffinose synthase, beta-fructofuranosidase, and 6-phosphate synthase were strongly expressed at 24 h in XS and at 6 h in GZ after cold stress (Figure 7B).

Moreover, most of the amino acid metabolites and their downstream products [including tryptophan (+), chlorogenate (−), swainsonine (−), and 2-methylmaleate (−)] showed higher levels in the two genotypes. In contrast, lysine (−) was upregulated in XS but downregulated in GZ under cold stress. LOC107850292 encoding a serine-pyruvate transaminase in serine metabolism was downregulated in GZ and XS after cold stress, while another gene (LOC107839921) encoding serine hydroxymethyltransferase was upregulated. This change in these two genes may be important for maintaining serine biosynthesis under cold stress. Interestingly, DEMs were not detected among the glycolysis and tricarboxylic acid cycle (TCA) metabolites under cold stress, whereas 9 and 10 DEGs were identified to be involved in these pathways, respectively (Figure 7B). The content of GSH increased in XS and decreased in GZ under cold stress. The content of glutamate and proline increased moderately after 6 h in XS, whereas they increased significantly in GZ after 24 h of cold stress. Two genes (LOC107838791 and LOC107850578) were predicted to encode 4-Aminobutanoic acid (GABA) showed higher levels in GZ under cold stress, especially at 6 h.

Polyamine synthesis can be divided into two pathways: ODC (LOC107866830) catalyzes ornithine to Put, and ADC (LOC107840486 and Newgene-15608) catalyzes arginine to Put. Furthermore, SPDS (LOC107847831) uses Put as a substrate to produce Spd and Spm. PAO (LOC107878076 and LOC107859655) can catalyze Spm to Spd ultimately leading to the formation of Put (Figure 7). All the above genes involved in polyamine metabolism were upregulated in the two genotypes under cold stress, and the genes in GZ were upregulated more than in XS at 6 h. In parallel with changes in the expression of genes, the activities of ADC, ODC, SPDS, and PAO also showed an increasing trend under cold stress. Still, there were significant differences, mainly at 24 h in XS and GZ (Supplementary Figure 5). Although the content of Put, Spd, and Spm increased in both genotypes, a greater increase was observed in the cold-resistant genotype than in the cold-sensitive genotype (Supplementary Figure 6).

## Discussion

With the frequent occurrence of extreme weather, climate has become a major factor affecting the high yield of crops. Multiple responses to cold stress in plants have been shown to reflect changes in transcription and metabolism (Zhao D. Q. et al., 2019; Raza et al., 2021; Sun et al., 2021). In this study, we identified 10,931 DEGs and 657 DEMs in positive ion mode and 390 DEMs in negative ion mode by multi-omics analysis. Furthermore, the DEGs and DEMs were involved in amino acid metabolism, PA metabolism, and signal transduction, which may play a significant role in response to cold stress.

### Osmotic Adjustment Under Cold Stress

Osmoregulation plays an important role in maintaining cell homeostasis in both normal and adverse environments. Several reports have shown that the accumulation of compatible solutes can maintain a stable osmotic pressure and protect functional proteins from degradation by mediating osmotic adjustment (Chen and Jiang, 2010; Turner, 2018). Our research showed that the levels of most sugars increased under cold stress in two cultivars, while some of them were recognized as osmolytes in the plants. Proline is an important amino acid for osmotic adjustment and is an important index for investigating cold tolerance responses in plants (Jia et al., 2016; Wang et al., 2018). Similar to previous studies, higher levels of proline were found in the cold-stressed plants than in the control. However, the proline content of the cold-sensitive cultivar (XS) decreased more sharply after the 24 h than after the 6 h treatment, indicating that the long cold stress period may lead to the osmotic disturbance causing, in turn, water loss and wilting (Figure 1A).

### Polyamine Metabolism Under Cold Stress

Polyamines are small aliphatic amines found in all living cells. Put, Spd, and Spm are major components in plants and play vital roles in plant growth, development, and stress response (Pál et al., 2015; Ghosh et al., 2021; Gonzalez et al., 2021). The levels of PAs increase considerably in response to cold stress (Amini et al., 2021; Jiao et al., 2021; Wang et al., 2021), and a cold-tolerant rice cultivar showed a greater accumulation of PAs than the sensitive cultivars (Sheteiwy et al., 2017). This study showed that the content of PAs increased in both genotypes under cold stress but the increase in GZ was higher than that in XS, suggesting that the changes in Put, Spd, and Spm levels play a key role in response to cold stress (Supplementary Figure 6). Furthermore, we investigated the changes in enzymes and genes related to polyamine metabolism. Many studies have indicated that ADC, ODC, SPDS, and PAO activities increase under cold stress (Diao et al., 2015; Zhao et al., 2017). Amini et al. (2021) reported that the expression of PAO, ADC, and SPDS genes in chickpea increased under cold stress, while the level of ODC gene decreased to some extent. In our study, the above gene levels and enzyme activities were upregulated in both species, with a more significant increase in GZ under cold stress at 6 h (Supplementary Figure 5). These results indicate that gene expression and enzyme activities were regulated earlier in the cold-tolerant cultivar (GZ) to improve cold tolerance.

### Major Signaling Pathways Under Cold Stress

**FIGURE 8:**
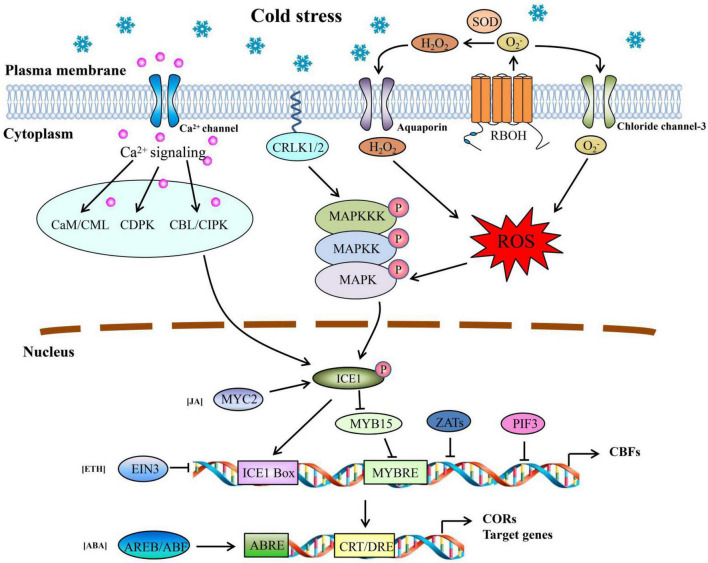
Model of the multiple signal transduction pathways and transcriptional networks during cold stress in pepper. Arrows represent positive regulation, whereas lines ending with a bar represent negative regulation.

Analysis of the transcriptome data and KEGG of DEMs showed that many genes were involved in signal perception, transduction, and regulation under cold stress. We constructed a network for signaling in response to cold stress in pepper based on these candidate genes encoding TFs, receptor proteins, and phosphorylation kinases (Figure 8). Several studies have reported that Ca^2+^ acts as a secondary messenger in response to cold stress and is recognized by Ca-binding proteins of mainly three categories: calmodulin/CaM-like proteins (CaM/CML), Ca^2+^ dependent protein kinases (CDPK), and calcineurin B-like proteins/calcineurin B-like protein-interacting protein kinases (CBL/CIPK; Guo et al., 2018; Zhao Y. et al., 2019). In this study, several genes were predicted to encode CML, CIPK, and CDPK-related kinases (CRK), and most of them were upregulated in response to cold stress (Supplementary Table 14). These genes may represent potential candidates for perceiving intracellular changes in Ca^2+^ levels and activating the expression of downstream COR genes in pepper.

Additionally, mitogen-activated protein kinase (MAPK) cascades, which contain MAP kinase kinase kinase (MAPKKK, or MEKK), MAP kinase kinase (MAPKK, or MKK), and MAP kinase (MAPK, or MPK), have been proposed to play a vital role in cold responses (Rodriguez et al., 2010). In *Arabidopsis*, there are two well-studied pathways, MEKK1-MKK2-MPK4 and MAP3K-MKK4/5-MPK3/6, regulated by calcium/calmodulin-regulated receptor-like kinase 1/2 (CRLK1/2; Furuya et al., 2013). Here, a total of 26 genes were predicted to encode MAPK-related proteins, mainly included 8 genes encoding MAPK proteins, 2 genes encoding MAPKK proteins, and 16 genes encoding MAPKKK proteins. Among them, a new gene (Newgene_9060) encoding MEKK3 was downregulated under cold stress, whereas two genes MKK4/5 (LOC107861622) and MPK3 (LOC107873437) were significantly upregulated under cold stress (Supplementary Table 14). Previous studies have shown that MKK4/5 plays a positive role in powdery mildew resistance, whereas MPK3/6 negatively regulates CBF expression in *Arabidopsis* under freezing stress (Zhao et al., 2014; Li et al., 2017).

Reactive oxygen species including, hydrogen peroxide (H_2_O_2_), superoxide anion (O_2_^–^), and hydroxyl radical (⋅OH−) are not only toxic by-products of metabolic processes but also act as signaling molecules in very different biological processes (Sierla et al., 2013; Wrzaczek et al., 2013; Jalmi and Sinha, 2015). H_2_O_2_ induced by heat stress can activate MAPK kinase, activating MPK3/6 in *Arabidopsis* (Kovtun et al., 2000). The N-terminal tails of Rbohs in plants contain two EF hands, which combine with Ca^2+^ and are necessary for Rboh activation (Ogasawara et al., 2008). Here, two genes were predicted to encode Rboh (LOC107864242 and LOC107862088) with more than six fold upregulation after 24 h of cold stress in both cultivars, which may involved in the ROS signaling pathway (Figure 8 and Supplementary Table 14). It has been shown that Rboh proteins transfer electrons from cytosolic flavin adenine dinucleotide (FAD) or nicotinamide adenine dinucleotide phosphate (NADPH) to apoplastic oxygen to form O_2_^–^, which is then converted to H_2_O_2_ by SOD (Wrzaczek et al., 2013). Kawarazaki et al. (2013) found that AtRbohF is activated by Ca^2+^ and protein phosphorylation and then binds to AtSRC12 to regulate ROS production. Of particular interest were the two genes (LOC107847894 and LOC107873309) predicted to encode SOD (including Cu/Zn-SOD and Fe-SOD) that were upregulated at 6 h but decreased significantly at 24 h in XS (Supplementary Table 14). This indicates that short-term cold stress could induce antioxidative defense mechanisms in plants, but when the antioxidative enzyme activity was impaired, the content of ROS increased and activated MAPK after a more extended stress period.

### Major Regulators and Regulatory Mechanisms Under Cold Stress

After signal perception and transduction, plants activate the expression of low-temperature responsive genes, which has been demonstrated to be critical for cold tolerance in plants. Lin and Thomashow (1992) first isolated a COR gene in *Arabidopsis*, named COR15, and identified the upstream regulatory factors CBF in the following years. CBF proteins recognize the CRT/DRE *cis*-element, which contains a conserved sequence (CCGAC) in the promoters of COR genes (Stockinger et al., 1997). Two CBF genes (CaCBF1A and CaCBF1B) induced by low-temperature stress were cloned from pepper (Kim et al., 2004). In this study, a CBF (LOC107863343, CaCBF1B) gene was upregulated under cold stress in both genotypes. However, this gene’s expression decreased sharply in XS after 24 h (Supplementary Table 14), suggesting that cold-resistant species (GZ) may continuously upregulate CBF expression to induce COR gene expression and improve cold resistance. The ICE, a positive upstream regulator of CBF, acts as a signal transducer related to cold stress in plants (Chinnusamy et al., 2003; Kim et al., 2004). However, the two genotypes showed no apparent differences in ICE1 and ICE1-like genes under cold stress. Therefore, it is necessary to understand the dynamic expression and function of ICE1 in pepper.

In addition, the expression of CBF genes was regulated by other regulators *via* specific *cis*-elements in the promoters. For example, MYB15, a member of the R2R3-MYB family protein, regulates CBF expression by binding to the MYB recognition element in CBF promoters under cold stress (Agarwal et al., 2006). Moreover, some C2H2-zinc finger proteins are also involved in the regulation of CBF expression. It has been shown that ZAT12-overexpressing-plants could inhibit the transcript levels of CBF genes (Vogel et al., 2005). The ZAT10 protein represses the expression of *RD29A*, a target gene of CBFs (Lee et al., 2002). In *the Arabidopsis* CBF1/2/3 triple mutant, ZAT10 expression was significantly repressed under cold stress (Zhao et al., 2016). Multiple MYB and MYB-related TFs and C2H2-zinc finger proteins were observed (Figure 4 and Supplementary Table 7). Interestingly, two genes (LOC107864180 and LOC107853100) encoding MYB15 protein showed opposing expression patterns, which may be related to its regulatory mechanism. Previous studies have shown that AtMYB15 acts as a negative regulator of CBFs in *Arabidopsis* (Agarwal et al., 2006). However, Zhang et al. (2020) found that the levels of MYB15 transcripts increased in tomatoes under cold stress (4°C), and in MYB15 mutations, the CBF transcript abundance decreased significantly. Kim et al. (2017) showed that MYB15 could interact with MPK6 to reduce its inhibitory effect on CBF gene expression. Moreover, PIFs (phytochrome-interacting factors), a subfamily of bHLH TFs, mainly participate in the photomorphogenic development, and several members also as thermo-sensors, involved in cold response (Jiang et al., 2020; Zheng et al., 2020). Yang et al. (2021) found that six PIF members were existed in pepper, including *CaPIF1*, *CaPIF3*, *CaPIF4*, *CaPIF7a*, *CaPIF7b* and *CaPIF8*, among them, *CaPIF8* was induced under cold stress. Here, a new gene (Newgene_7768) encoding PIF3 was identified and was downregulated under cold stress (Supplementary Table 14), which was consistent with the present results on PIF3 (Jiang et al., 2017). In the future, it is interesting to understand the mechanism of this new gene in response to cold stress.

Similar to our studies on plant hormone metabolism (Figure 6), some studies have reported that several intermediates are involved in regulating the ICE-CBF-COR pathway (Figure 8). For example, MYC2 is an activating TF in the response pathway of JA in plants and interacts with ICE1 to increase the transcription of CBF genes. Wang et al. (2019) proposed that the overexpression of *MdMYC2* in apples enhanced the expression of *MdCBF* genes, resulting in increased cold tolerance. In ETH signal transduction, EIN3 can directly bind to the promoters of *CBF* genes to repress their expression (Jiang et al., 2017). ABA is a well-studied hormone involved in regulating cold stress and is known as the ABA-dependent pathway. BA-responsive elements (ABREs) are major *cis*-acting elements in CORs or other target genes (ABA-responsive genes) (Huang et al., 2012; Shi and Yang, 2014). In *Arabidopsis*, approximately 10% of cold-induced genes contain CRT/DRE and ABRE elements in their promoter regions (Shi and Yang, 2014). The ABRE-binding protein/ABRE-binding factor (AREB/ABF) and bZIP TFs can bind to ABRE and activate target gene expression. Several genes encoding these three proteins (MYC2, EIN3, and AREB/ABF) were observed, and most were upregulated (Figure 6 and Supplementary Table 14). Moreover, the contents of ABA and JA increased significantly in cold-resistant cultivars (Figure 6), indicating that high levels of ABA and JA may participate in the responses of pepper to cold stress through the ICE-CBF-COR pathway.

In summary, the regulation of cold stress in plants is a complex process. We mainly focused on the ICE-CBF-COR pathway. Many other pathways are involved in the regulation of cold stress. In addition, the function of each candidate gene needs to be confirmed by different methods. Therefore, further work is required to elucidate the mechanism of cold stress in pepper plants.

## Conclusion

This study comprehensively analyzed the regulation mechanism in two pepper genotypes with contrasting cold tolerance using transcriptomics and metabolomics. It was found that free PAs and osmolytes, such as putrescine, spermine, spermidine, raffinose, and proline, play important roles in regulating cold stress in pepper. Moreover, the regulation of the ICE-CBF-COR pathway by TFs and plant hormones may explain cold tolerance of GZ. Our study provides insights into the cold tolerance mechanisms of pepper and a valuable foundation for pepper breeding.

## Data Availability Statement

The original contributions presented in the study are publicly available. This data can be found here: National Center for Biotechnology Information (NCBI) BioProject database under accession number PRJNA778231.

## Author Contributions

JZ and HL conceived and designed the experiments. JZ, LL, ZZ, and LS performed the experiments. JZ, YX, LS, YT, and YL analyzed the data. JZ wrote the manuscript. JZ, YT, BS, and HL reviewed and revised the manuscript. All authors have read and approved the final version of the manuscript.

## Conflict of Interest

The authors declare that the research was conducted in the absence of any commercial or financial relationships that could be construed as a potential conflict of interest.

## Publisher’s Note

All claims expressed in this article are solely those of the authors and do not necessarily represent those of their affiliated organizations, or those of the publisher, the editors and the reviewers. Any product that may be evaluated in this article, or claim that may be made by its manufacturer, is not guaranteed or endorsed by the publisher.

## Footnotes

1
https://www.ncbi.nlm.nih.gov/genome/10896

2
http://www.biocloud.net/

3
http://primer3.ut.ee/
