# Corrigendum: Transcriptome and Metabolome Analyses Reveal Molecular Responses of Two Pepper (*Capsicum annuum* L.) Cultivars to Cold Stress

**DOI:** 10.3389/fpls.2022.975330

**Published:** 2022-07-06

**Authors:** Jianwei Zhang, Le Liang, Yongdong Xie, Zhao Zhao, Lihong Su, Yi Tang, Bo Sun, Yunsong Lai, Huanxiu Li

**Affiliations:** ^1^College of Horticulture, Sichuan Agricultural University, Chengdu, China; ^2^Institute for Processing and Storage of Agricultural Products, Chengdu Academy of Agricultural and Forest Sciences, Chengdu, China; ^3^Institute of Pomology and Olericulture, Sichuan Agricultural University, Chengdu, China

**Keywords:** pepper, transcriptomic, metabolome, cold stress, polyamines, ICE-CBF-COR

In the published article, there was an error in Supplementary Figure 6. We used the wrong formula in the determination of polyamine (Put, Spd, Spm) content. The correct material statement appears below.

**Supplementary Figure 6:**
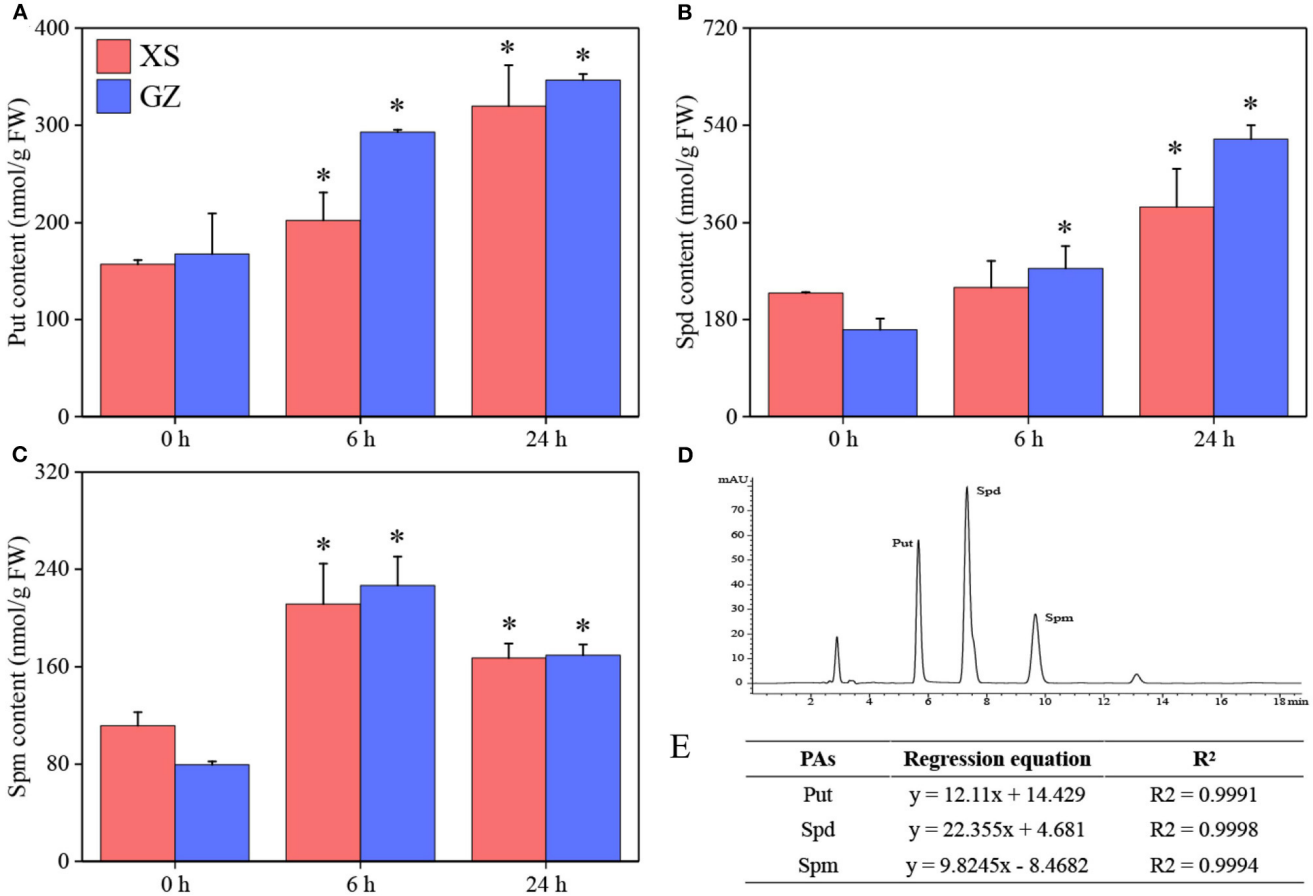
Determination of PAs contents in XS and GZ under cold stress. **(A)** Put content. **(B)** Spd content. **(C)** Spm content. **(D)** Chromatogram of mixed standard solution of PAs. **(E)** The regression equation and correlation coefficient of PAs.

The authors apologize for this error and state that this does not change the scientific conclusions of the article in any way. The original article has been updated.

## Publisher's Note

All claims expressed in this article are solely those of the authors and do not necessarily represent those of their affiliated organizations, or those of the publisher, the editors and the reviewers. Any product that may be evaluated in this article, or claim that may be made by its manufacturer, is not guaranteed or endorsed by the publisher.

