# Supplementary material for: Transcriptome and Metabolome Analyses Reveal Molecular Responses of Two Pepper (Capsicum annuum L.) Cultivars to Cold Stress
Source: Front Plant Sci. 2022 Mar 22;13:819630. doi: 10.3389/fpls.2022.819630 (PMC8981722; doi:10.3389/fpls.2022.819630)
Supplement: Supplementary file 15 [file Data_Sheet_1.docx]

Supplementary Material

# Supplementary Figures
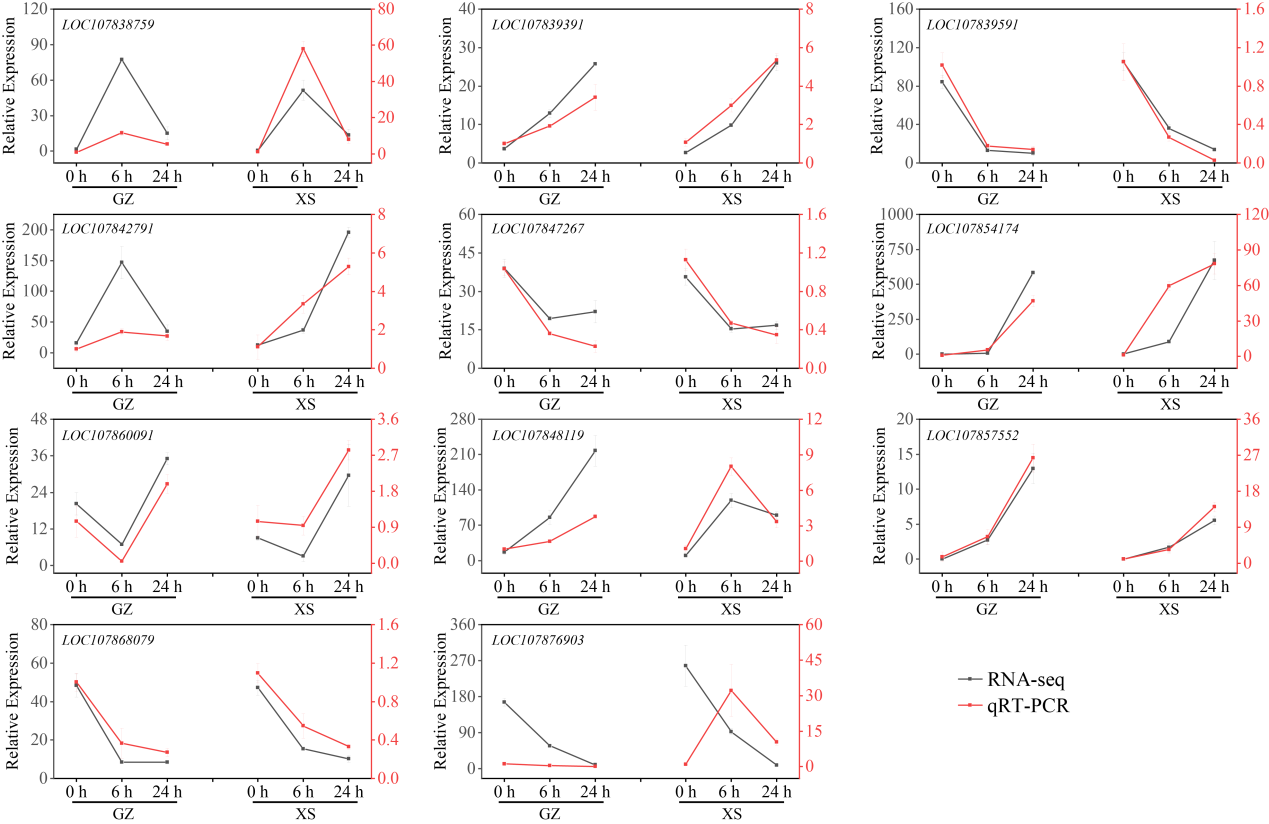


**Supplementary Figure 1.**Validation by qRT-PCR of DEGs isolated from the different samples in ‘XS’ and ‘GZ’.


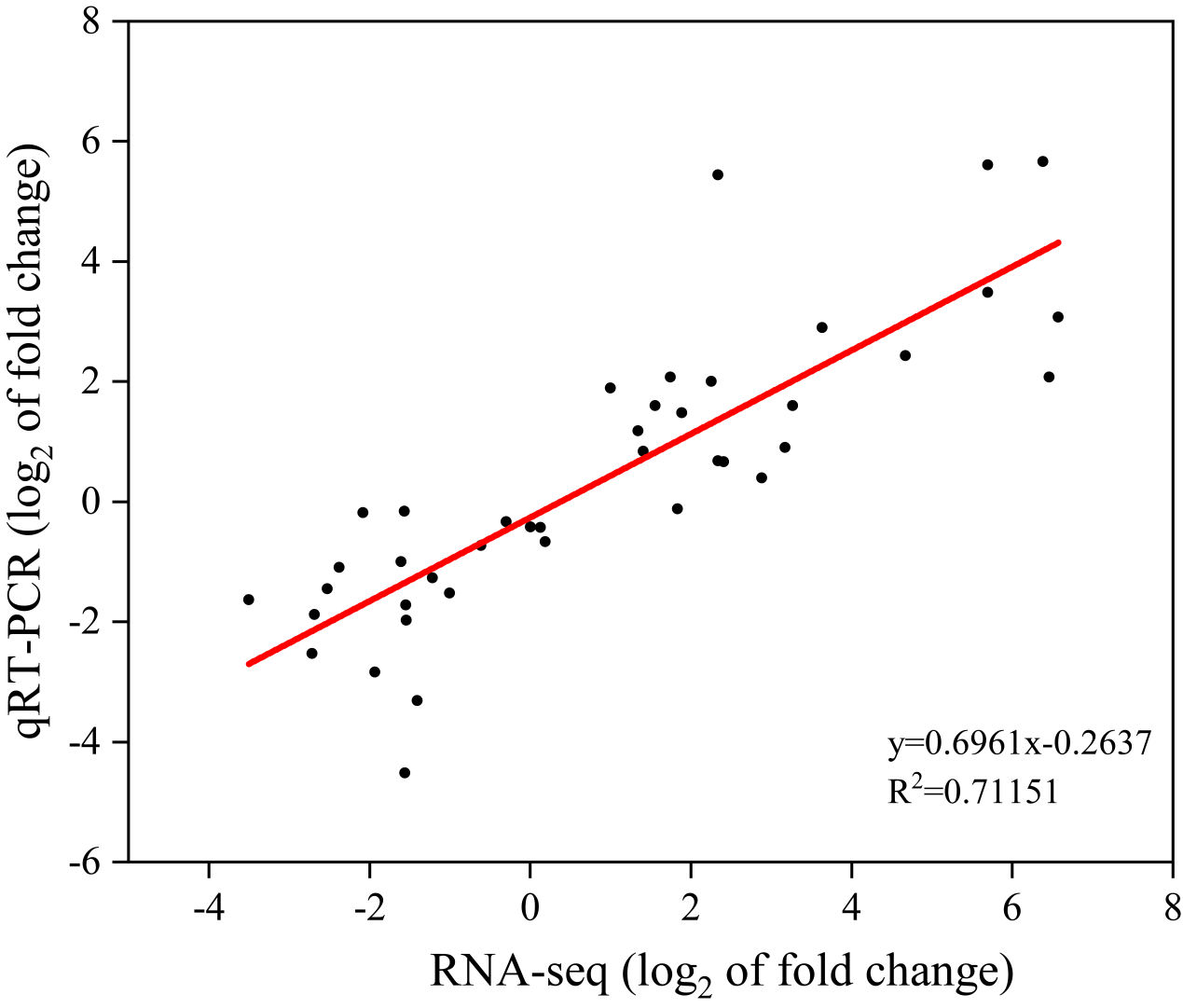


**Supplementary Figure 2.**Correlation between RNA-Seq expression profile and qRT-PCR results.


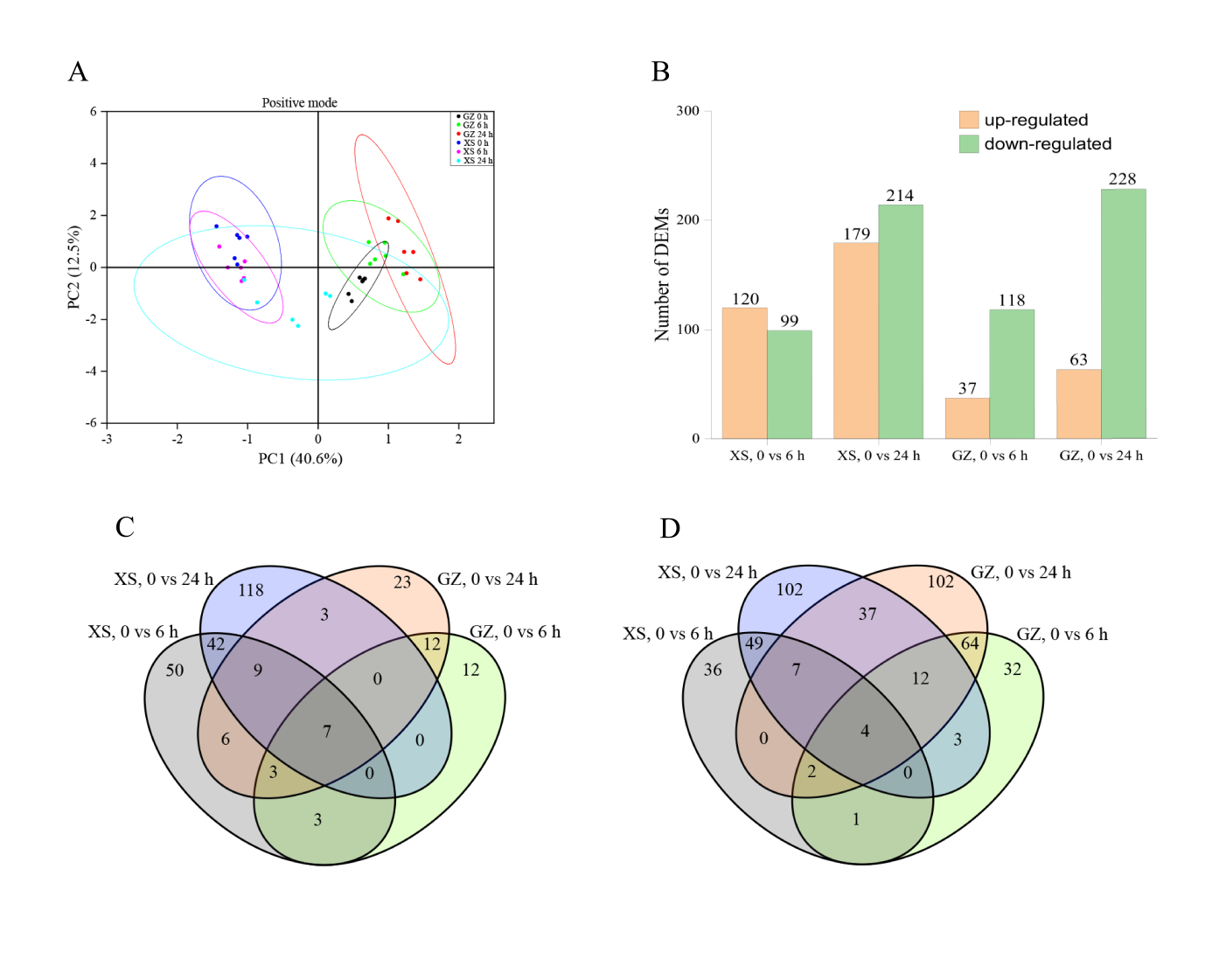


**Supplementary Figure 3.** The analysis of DEMs in positive ion mode. **(A)**Principal component analysis (PCA) of metabolic profiles. (**B**)The total number of up-regulated and down-regulated DEMs. **(C)** Venn diagram of up-regulated DEMs. **(D)** Venn diagram of down-regulated DEMs.


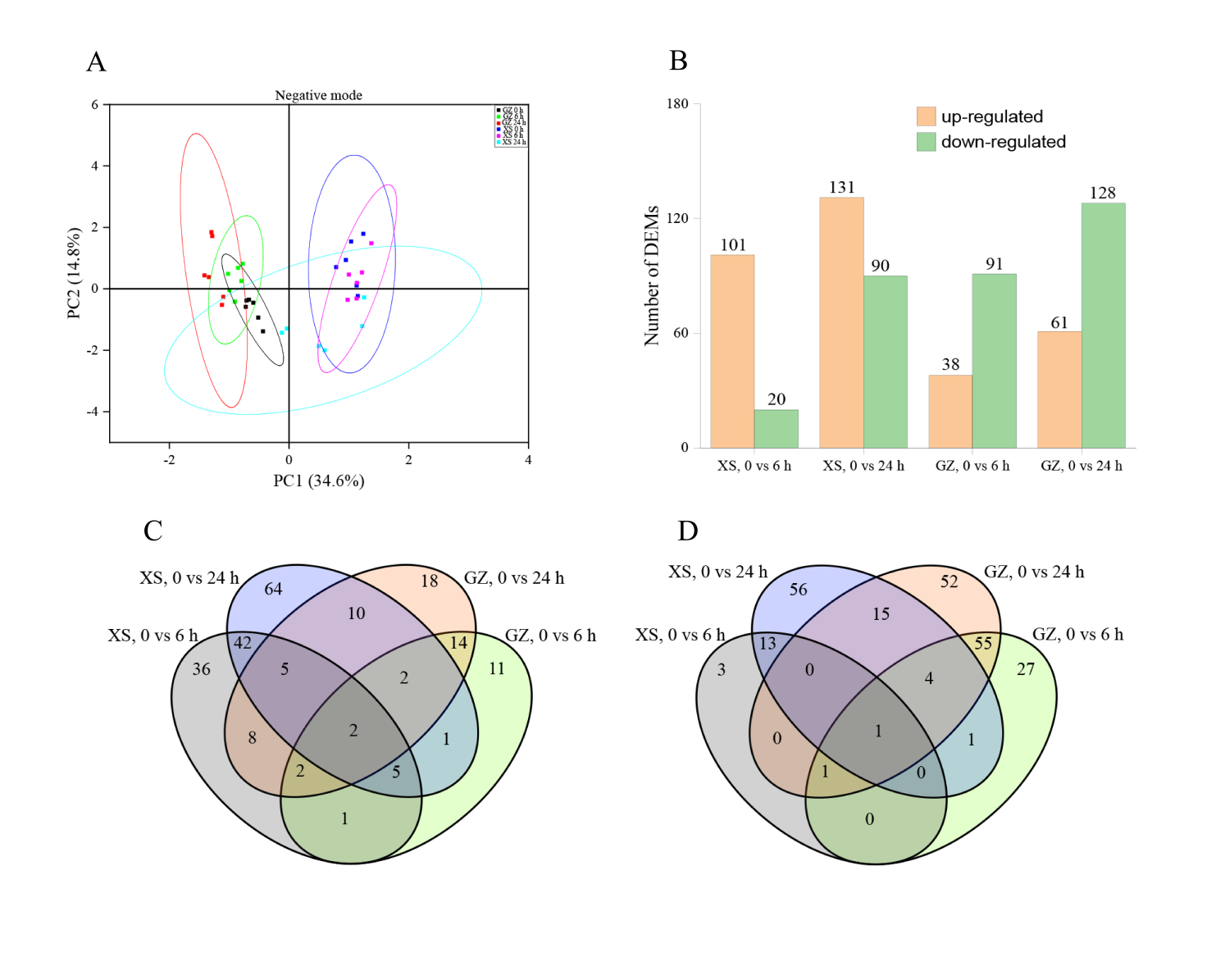


**Supplementary Figure 4.** The analysis of DEMs in negative ion mode. **(A)**Principal component analysis (PCA) of metabolic profiles. **(B)**The total number of up-regulated and down-regulated DEMs. **(C )**Venn diagram of up-regulated DEMs. **(D)** Venn diagram of down-regulated DEMs.


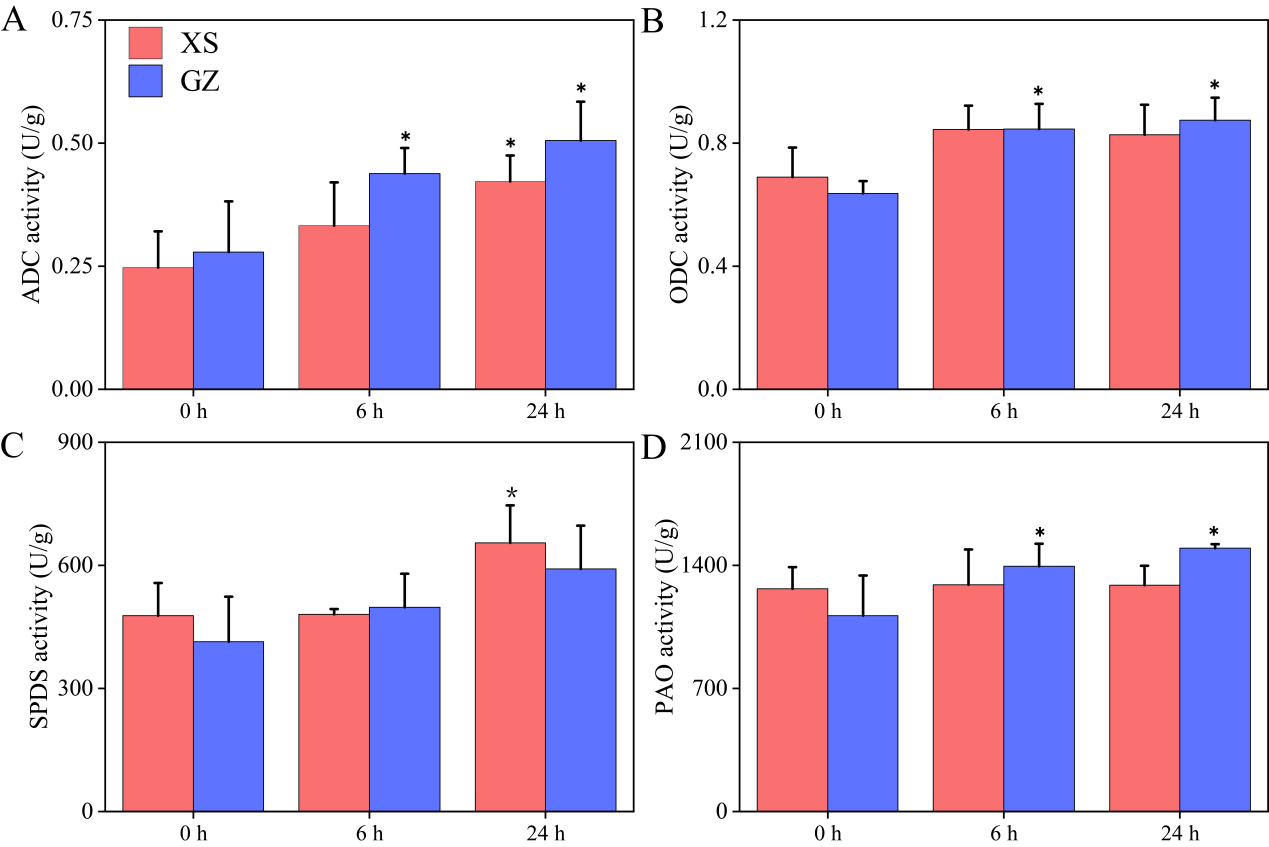


**Supplementary Figure 5.** Determination of enzyme activities in XS and GZ under cold stress. **(A)** ADC activity. **(B)** ODC activity. **(C)** SPDS activity. **(D)** PAO activity.


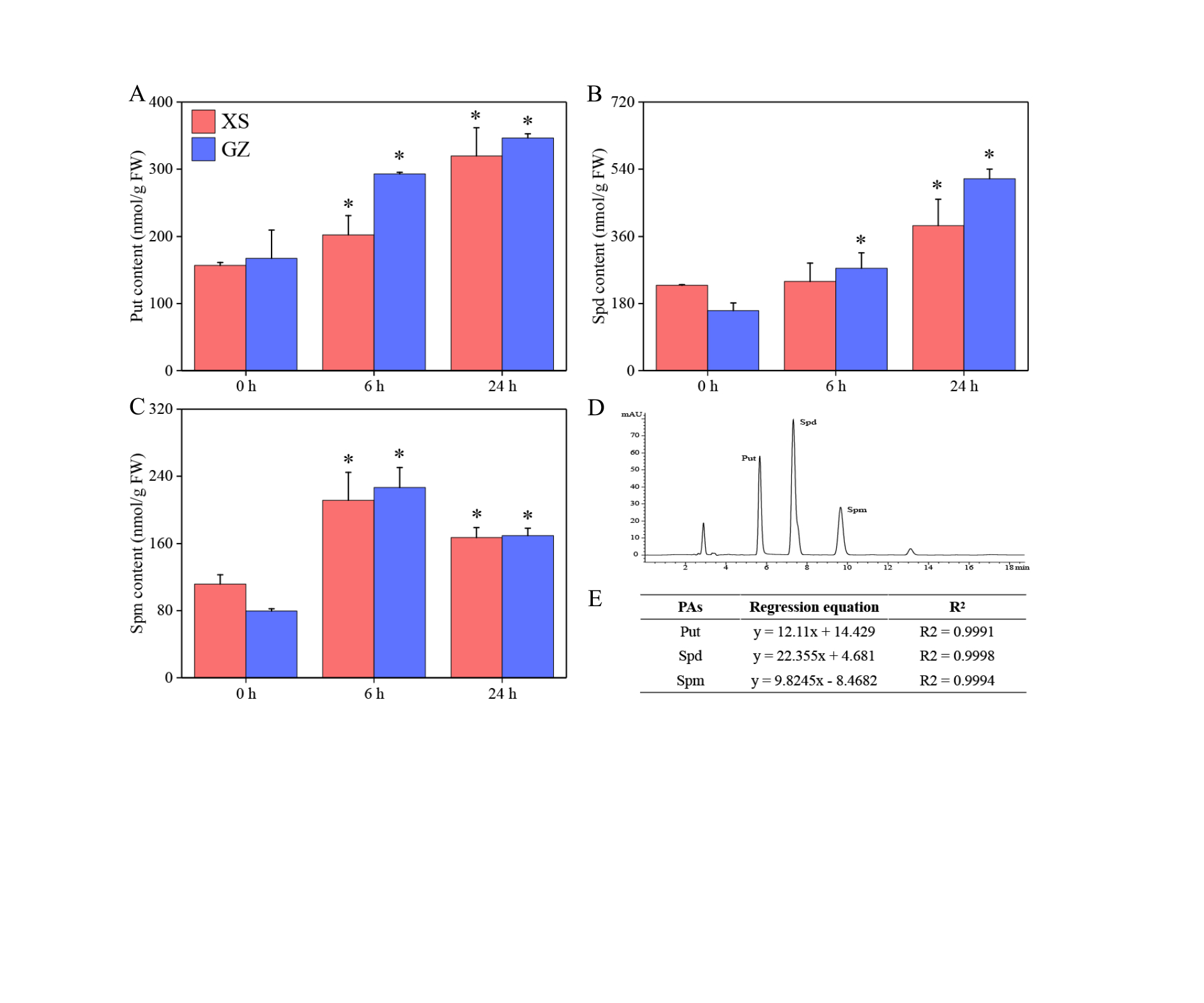


**Supplementary Figure 6.** Determination of PAs contents in XS and GZ under cold stress. **(A)** Put content. **(B)** Spd content. **(C)** Spm content. **(D)** Chromatogram of mixed standard solution of PAs. **(E)** The regression equation and correlation coefficient of PAs.
